# Novel Capacitive Sensing System Design of a Microelectromechanical Systems Accelerometer for Gravity Measurement Applications

**DOI:** 10.3390/mi7090167

**Published:** 2016-09-14

**Authors:** Zhu Li, Wen Jie Wu, Pan Pan Zheng, Jin Quan Liu, Ji Fan, Liang Cheng Tu

**Affiliations:** 1 MOE Key Laboratory of Fundamental Physical Quantities Measurement, School of Physic, Huazhong University of Science and Technology, Wuhan 430074, China; lizhu@hust.edu.cn (Z.L.); D201177044@hust.edu.cn (W.J.W.); xingruiyuan@hust.edu.cn (P.P.Z.); jinquanliu@hust.edu.cn (J.Q.L.); fanji@hust.edu.cn (J.F.); 2 Institute of Geophysics, Huazhong University of Science and Technology, Wuhan 430074, China

**Keywords:** MEMS, three dimensional (3D), capacitive sensing system design, large dynamic range, high resolution, high-precision measurement

## Abstract

This paper presents an in-plane sandwich nano-g microelectromechanical systems (MEMS) accelerometer. The proof-mass fabrication is based on silicon etching through technology using inductive coupled plasma (ICP) etching. The capacitive detection system, which employs the area-changing sensing method, combines elementary capacitive pickup electrodes with periodic-sensing-array transducers. In order to achieve a large dynamic range with an ultrahigh resolution, the capacitive detection system employs two periodic-sensing-array transducers. Each of them can provide numbers for the signal period in the entire operating range. The suspended proof-mass is encapsulated between two glass caps, which results in a three dimensional structure. The measured resonant frequency and quality factor (*Q*) are 13.2 Hz and 47, respectively. The calibration response of a ±0.7 g input acceleration is presented, and the accelerometer system presents a sensitivity of 122 V/g and a noise floor of 30 ng/√Hz (at 1 Hz, and 1 atm). The bias stability for a period of 10 h is 30 μg. The device has endured a shock up to ±2.6 g, and the full scale output appears to be approximately ±1.4 g presently. This work presents a new opportunity for highly sensitive MEMS fabrication to enable future high-precision measurement applications, such as for gravity measurements.

## 1. Introduction

After more than two decades of research and development, microelectromechanical systems (MEMS) accelerometers have been applied widely in the automobile industry and in the field of consumer electronics [1,2]. Nowadays, more and more researchers are focused on developing three-dimensional (3D) MEMS, flexible MEMS, wearable MEMS, and high-precision MEMS [3,4,5,6,7]. In contrast with traditional quartz-flexure accelerometers and spring accelerometers, which have already been used in commercial high-precision (nano-g resolution where 1 ng = 9.8 × 10^−9^ m/s^2^) measurement instruments, such as a rotating accelerometer-based gravity gradient instrument (GGI), high-precision MEMS accelerometers present the potential benefits of low cost, small form factor, and low power dissipation [8]. Moreover, many high-precision measurements make use of the difference between the outputs of pairs of linear accelerometers, which results in cancelling out the common mode accelerations caused by the mounting platform and external environment. In this case, one of the key technologies is to match the acceleration-to-voltage or acceleration-to-current transfer functions of the pairs of the accelerometers to an extremely high degree of accuracy [9]. Unlike a non-MEMS accelerometer, the uniformity of MEMS fabrication (wafer-to-wafer or run-to-run) is relatively high. This high uniformity can effectively reduce the difficulty of matching. In addition, since the crystal lattice arrangement is very well-controlled in single crystal silicon, the mechanical properties of the silicon-based spring are very stable. Table 1 compares the mechanical properties of signal crystal silicon with various other materials that can be used as the spring in accelerometer fabrication. The thermal and fatigue resistance of a silicon-based spring are also better than those of a metal spring. Although quartz presents a better coefficient of thermal expansion, the micromachining of quartz is more difficult than that of silicon. This drawback limits its development and application for 3D integration.

Although earlier literature has already addressed the challenges of ultra-high resolution MEMS acceleration sensors [6,15], some subwavelength optical gratings need to be integrated into MEMS devices or a complex external optical detection system must be conjugated during field application. Recently, 3D integration technology has been widely recognized as the next generation of semiconductor manufacturing technology [16,17]. Although some heterogeneous integration methods, such as epitaxy [18] and bonding [19], have been reported, the high-quality integration of III-V compound semiconductors on a non-lattice-matched silicon substrate is a major concern in silicon photonic devices. Hence, MEMS and complementary metal-oxide-semiconductor (CMOS) integration was the primary choice using monolithic and hybrid/package approaches until recently [20]. Compared with other technologies, such as tunneling current [21,22] and laser interferometry [23,24], capacitive MEMS accelerometers are very suitable for future 3D heterogeneous integration.

**Figure 1:**
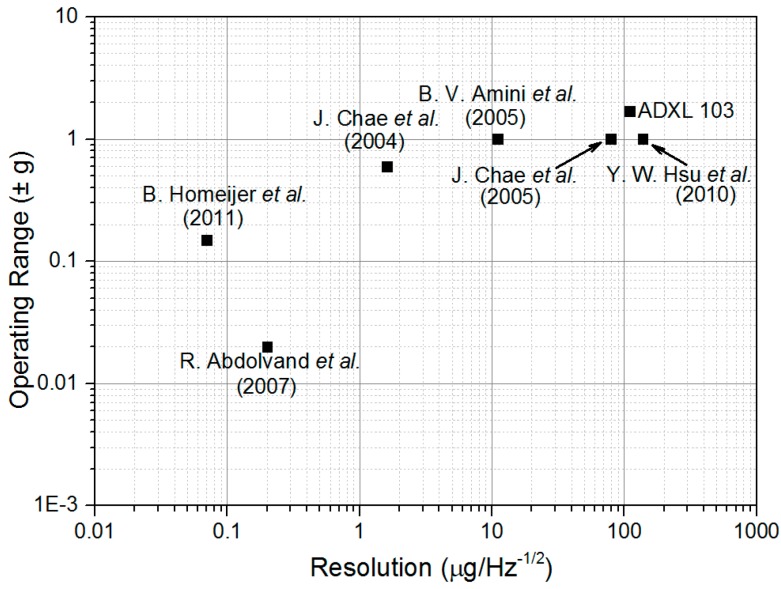
Performance of some reported in-plane MEMS accelerometers.

In order to obtain an extra-high resolution for this kind of MEMS accelerometer, some researchers have used full-wafer thickness to increase the proof-mass weight [25,26,27]. The noise can be effectively reduced, and the sensitivity significantly improved. However, the results demonstrated in the literature are not suitable for some low-frequency measurements, such as high-precision gravity or gradient measurements, since the reported ultrahigh resolutions are obtained at a frequency higher than 1 Hz. For instance, for rotating accelerometer-based gravity gradient instrument (GGI) applications, the rotor rotates at a low speed (<1 Hz), typically 0.25 Hz [28,29] or 0.1 Hz [9], which requires high resolution at the corresponding frequencies. Figure 1 summarizes the noise performance (overall accelerometer system noise) and the operating range of some reported in-plane capacitive MEMS accelerometers [8,25,26,30,31,32]. In order to achieve an extra-high resolution up to nano-g, some researchers chose to compromise on operating range [25,26]. If the requirement of operating range reaches ±1 g, the noise floor was usually in the level of tens of micro-g [30,31,32]. In particular, owing to the 1/*f* noise, the noise floor was often very high at a low frequency. Hence, it is very difficult to achieve a high accuracy in-plane MEMS accelerometer at a low frequency with a large operating range.

In this paper, we detail a capacitive sensing design for a MEMS accelerometer meeting nano-g resolution requirements with a large operating range at lower frequencies (0.1–1 Hz). The detection system consists of two periodic-sensing-array transducers, which are used to achieve a high sensitivity by subdividing the operating range. Combining these transducers with elementary capacitive pickup electrodes, a large dynamic range can be obtained. The MEMS fabrication is accomplished by using silicon through etching technology. The calibration results in a large operating range of ±0.7 g are presented. The sensitivity and the system resolution are 122 V/g and 100 ng/√Hz at 0.1 Hz (1 atm), respectively. A lower output noise floor is obtained from 0.1 Hz to 1 Hz (30 ng/√Hz at 1 Hz). The device has endured a shock up to ±2.6 g, and the full scale output appears around ±1.4 g. This nano-g resolution at a low frequency is very attractive for gravity measurement applications.

## 2. Capacitive Detection System Design

Depending on the sensing mode, the design can be divided into two types: variable area and variable gap capacitance sensing. Generally, in consideration of the differential output, the latter presents better sensitivity. However, in some applications, the poor sensing linearity and the small dynamic range of this sensing mode are usually identified as the bottleneck. To improve resolution, the simplest approach is to enhance output variation corresponding to a certain displacement, which means the slope of the output must have a greater absolute value. However, it is impractical to achieve a very steep output increase (or decrease) in a large operating range. Recently, Pike et al. [7,33] proposed a remarkable capacitive nano-g MEMS seismometer design. The researchers employed a rough linear detector and a fine periodic array transducer as the capacitive detection system. The rough detector is used for an approximate position determinant of the proof-mass in the operating range. The fine periodic array transducer gives a sinusoidal output, which could provide an ultrahigh resolution in a narrow band. By combining these two signals, the sensor can detect a nano-g signal in the entire operating range. Their latest device was used in the InSight mission to Mars. It can measure the vibration variation down to 2 ng/√Hz [34]. Its operational range is designed for a seismometer application, making it insufficient for gravity measurements.

**Figure 2:**
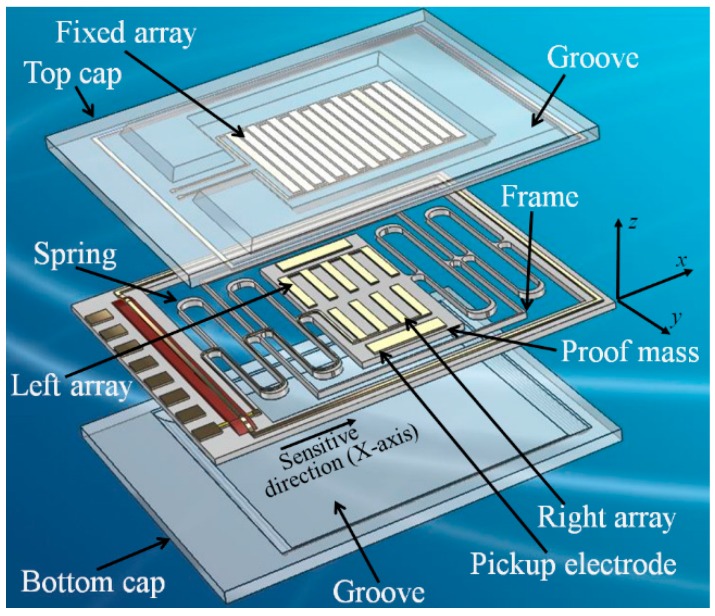
Perspective view of the designed capacitive sandwich structure MEMS accelerometer.

**Figure 3:**
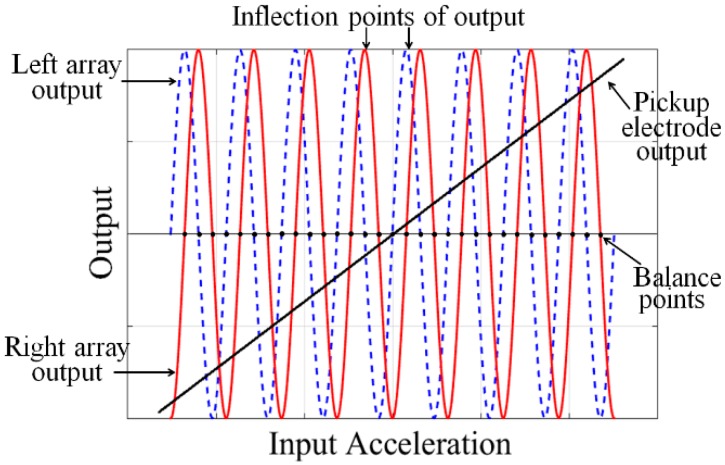
Theoretical output of MEMS accelerometer with two alternate array transducers.

Guided by this excellent design, two parallel alternating periodic-sensing-array transducers are introduced in the detection system to satisfy the critical requirement of a high precision MEMS accelerometer with a large operating range. Figure 2 shows a schematic view of our MEMS accelerometer. The MEMS consist of three parts: a top cap, a middle suspended proof-mass, and a bottom cap. Figure 3 depicts the theoretical outputs from the alternating array transducers. Three differential outputs can be detected. A rough linear output is provided by the elementary capacitive pickup electrodes, while the two sinusoidal outputs are the signals detected by the left and right periodic-sensing-array transducer. The designed phase difference of the sinusoidal outputs is π/2. Compared to MEMS with a single array transducer, two alternating array transducers make a further subdivision in operating range, which can effectively avoid the insensitive region when using single transducer. Although there are numbers of inflection points for each array transducer output, the two alternate array outputs ensure that at least one array output can work in the most sensitive area. Reading the output in order, a high resolution can thus be achieved in the entire operating range for an open-loop system. If the device is used in a close-loop system, the MEMS would work around the balance points of the sinusoidal signals. As shown in Figure 3, two sinusoidal outputs provide more balance points. Since the dynamic range around each balance point becomes smaller, the required strength of the feedback current can be reduced. Pursuing this further, smaller dynamic range around each balance point reduces the stability and uniformity requirements of the magnetic field in GGI applications. Therefore, closed-loop control can easily be achieved.

**Figure 4:**
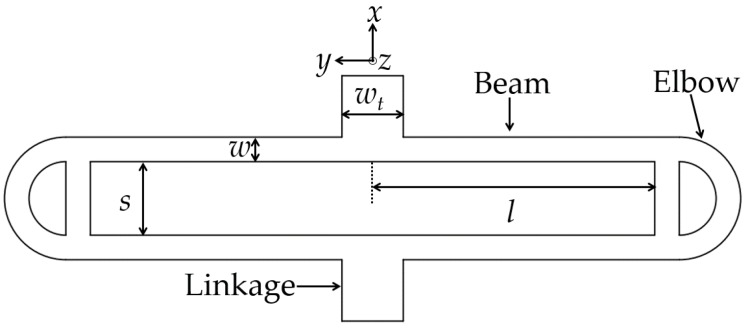
Schematic of a single folded spring set.

Some geometrical design details of our MEMS are listed in Table 2. In order to reduce the cross sensitivity, a symmetrical sandwich structure is designed. A large 18 mm × 15 mm proof-mass is used to decrease the mechanical noise. Employing a 500-μm-thick silicon substrate, the mass of the proof-mass can reach 0.31 g. The schematic of a single folded spring set is shown in Figure 4. A reasonable compromise between good stiffness and *z*-axis deflection can be achieved using a semicircular elbow with an unfilled cross bar spring design [5].

**Figure 5:**
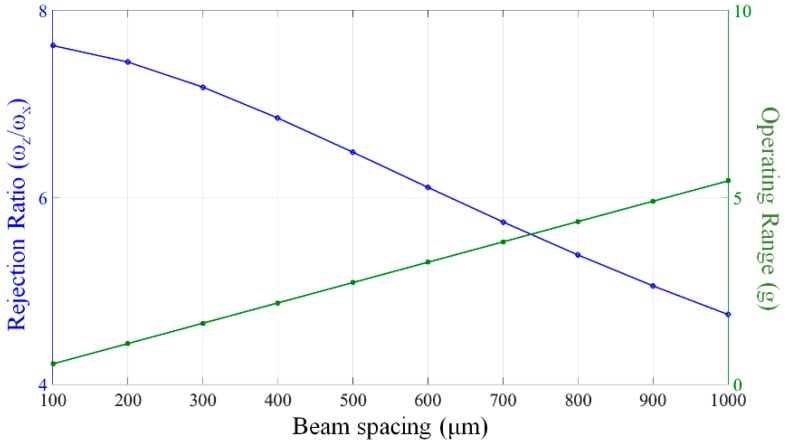
Rejection ratio of z-mode to the fundamental mode and the operating range with varying beam spacing.

It is well-known that, when the operating frequency is much smaller than the resonant frequency, the mechanical sensitivity *S* can be identified by

(1) $$S=\frac{m}{k}=\frac{1}{\omega_{0}^{2}}=\frac{\Delta x}{\Delta a}$$

where ω_0_ is the fundamental angular resonant frequency, Δ*x* is the displacement of the mass in the sensitive direction, and Δ*a* is the acceleration in the sensitive direction. It can be observed that the measured acceleration can be enlarged by increasing the fundamental resonant frequency. However, the mechanical noise of the accelerometer *NEA_mechanical_* is given by [35]

(2) $$NEA_{mechanical}^{2}=4k_{b}T\frac{\omega_{0}}{mQ}$$

where *k_b_* is the Boltzmann constant, *T* is the operating temperature and *Q* is the quality factor of the accelerometer. It is apparent from this equation that the mechanical noise will become larger while increasing the resonant frequency. Therefore, the operating range can be increased by enlarging the displacement of the mass in the sensitive direction. This seems to be an appropriate method for our application. In this case, six folded springs located at each side of the proof mass are used to obtain an operating range larger than 1 g. The stiffness of our spring design is 2.9 N/m. Moreover, this series design of spring can effectively minimize the resonant frequency, thus reducing the mechanical noise of our MEMS. However, more series springs decrease the rejection ratio of the spurious mode to the fundamental mode, especially the mode perpendicular to the sensing axis (z-mode). Since we employ the area changing sensing method for the *x*-axis, the stability of the distance in the *z*-axis direction will be a major concern in order to achieve high resolution. Moreover, the deflection in the out-of-plane *z*-direction is usually the second vibrational mode, which is the most important deflection to be minimized. According to the literature [7,33], five intermediate frames are introduced into the folded springs to increase the cross-axis rigidity and protect fragile structures in case of overload. The beam spacing (*s*, shown in Figure 4) of the folded spring is also carefully optimized to increase the rejection ratio. The rejection ratio for the z-mode is given by

(3) $${\left(\frac{\omega_{z}}{\omega_{0}}\right)}^{2}=\frac{1}{\frac{3EI_{x}s^{2}}{GJl^{2}}+\frac{2cI_{x}}{I_{z}}}$$

where ω*_z_* is the resonant frequency for the z-mode, *E* is the Young’s modulus of silicon, *c* is the elbow compliance factor (*c* = 1.2 in our design [5]), *I_x_* is the area moment of inertia about the *x*-axis, *G* is the modulus of rigidity, *J* is the torsional constant for spring beam about the *z*-axis and *I_z_* is the area moment of inertia about the *z*-axis. In order to minimize the mechanical noise and increase the operating range, we need to enlarge the beam spacing. However, it is apparent from (3) that the rejection ratio for the z-mode becomes worse while increasing the beam spacing. Figure 5 shows the rejection ratio of the z-mode to the fundamental mode and evolution of operating range as a function of beam spacing (using six folded springs for each side of the proof mass). It can be observed that the rejection ratio and the operating range need to be balanced during design. Finally, the beam spacing is chosen to be 480 μm, which could satisfy the requirements of rejection ratio and operating range in our design. The overall dimension of the suspended die is 44.5 mm × 32.2 mm.

**Figure 6:**
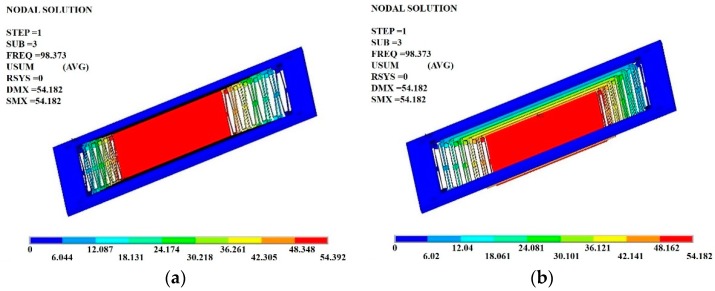
FEM simulation results of the vibration mode. (**a**) Fundamental mode that the vibration in the sensing axis; (**b**) mode perpendicular to red plane on the mass (z-mode).

To increase the survivability, the proof-mass movement can be constrained by some shock stops and dampers when the acceleration is overloaded in the sensing axis. Using the finite-element-method (FEM) software ANSYS (ANSYS, Inc., Canonsburg, PA, USA), the mechanical characterizations are calculated by numerical simulation (Figure 6). The dynamic analysis of the suspended structure shows that the resonant frequency for the fundamental mode corresponds to a vibration in the sensing axis with a frequency of about 15.2 Hz. The resonant frequency corresponds to the mode perpendicular to the sensing axis with a frequency of about 98.4 Hz. The other spurious modes resonant frequencies are also much higher (>92 Hz) than the fundamental resonant frequency, which means the sensing mode is successfully decoupled from other vibration modes. Thus, the accelerometer is only sensitive to the horizontal direction (*x*-axis).

## 3. Fabrication

**Figure 7:**
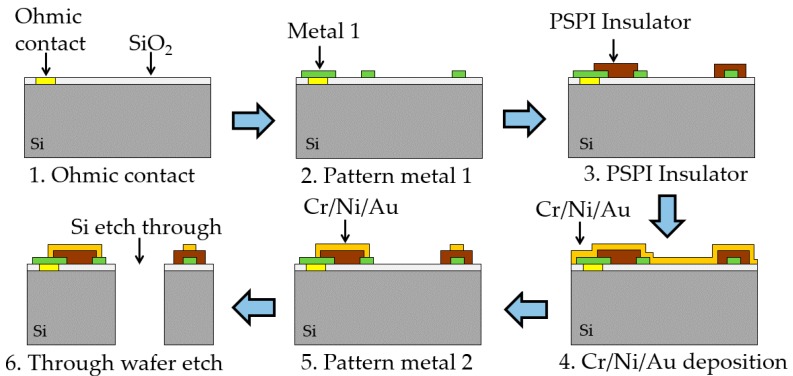
Schematic illustration and process flow for the complete suspension fabrication with metal and insulator layers.

**Figure 8:**
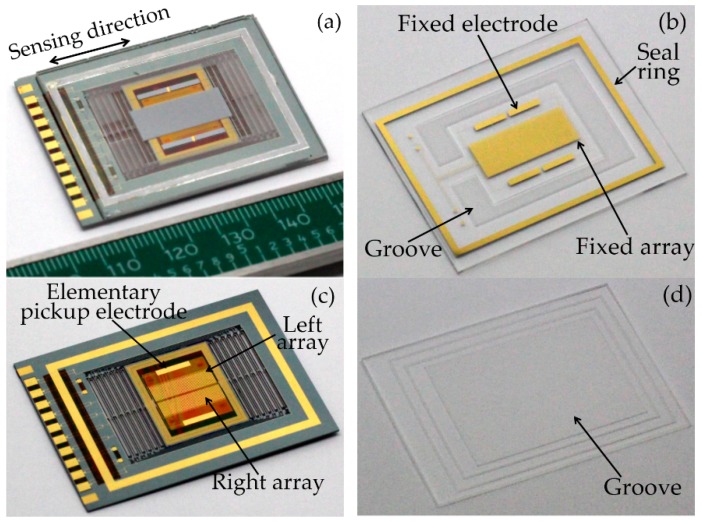
Encapsulation of MEMS accelerometer: (**a**) Assembled MEMS accelerometer; (**b**) top cap; (**c**) suspended proof-mass; (**d**) bottom cap.

Figure 7 schematically shows the process flow for the MEMS accelerometer fabrication. Silicon wafers (4 in, double polished, thickness 500 µm, *n*-type, (100) orientation, resistivity 0.01–0.09 Ω·cm, total thickness variation (TTV) <7 µm) with a 200-nm thermal oxide layer are used in this fabrication. After SiO_2_ etching by HF, the AuSb (1% Sb, 150 nm)/NiCr (50 nm)/Au (200 nm) metal stack is patterned. Subsequently, the wafer is annealed at 420 °C for 1 h for ohmic contact formation (step 1). Antimony acts as a shallow donor for ohmic contact on *n*-type Si [36]. NiCr is used as a barrier and an adhesion layer between AuSb layer and Au layer. A contact resistivity of 10^−3^ Ω·cm^2^ is achieved. In order to form the ground-plane for capacitive shielding and interconnection between different pads, a 20-nm Cr adhesion layer and a 200-nm Au metal layer are deposited (step 2). Next, a layer of photosensitive polyimide (PSPI) is spin coated and defined photolithographically to insulate the metal 1 layer from the sensing electrodes on top (step 3). This insulating layer was hardened by exposure to air at 80 °C for 120 min + 150 °C for 60 min + 180 °C for 60 min + 250 °C for 60 min + 350 °C for 60 min in a dry furnace for the purpose of imidization [37]. Our earlier studies indicate that PSPI is very suitable as an insulating application, and there is no risk of compatibility in following fabrication processes [37,38]. Further, a thin layer of Cr (20 nm)/Ni (80 nm)/Au (300 nm) is deposited (step 4). It is used for the topmost metal 2 layer that forms the sensing electrodes, encapsulation rings, traces and pads (step 5). Ni is employed to prevent dewetting effects during the encapsulation process. Then, an inductively coupled plasma (ICP) etching through process is performed to obtain the suspended proof-mass (step 6). Two caps made of borosilicate glass are employed for the final MEMS encapsulation. In order to reduce the damping effect, sand powder blasting is performed to form grooves on both caps. The sensing structure and packaging seal ring are patterned on the top cap. Finally, the top and bottom caps are attached to the proof-mass using SnPb solder bonding and organic glue, respectively (Figure 8). To increase the sensitivity, the distance between the proof mass and the top cap is controlled at 20 ± 3 μm.

## 4. Characterization Results

**Figure 9:**
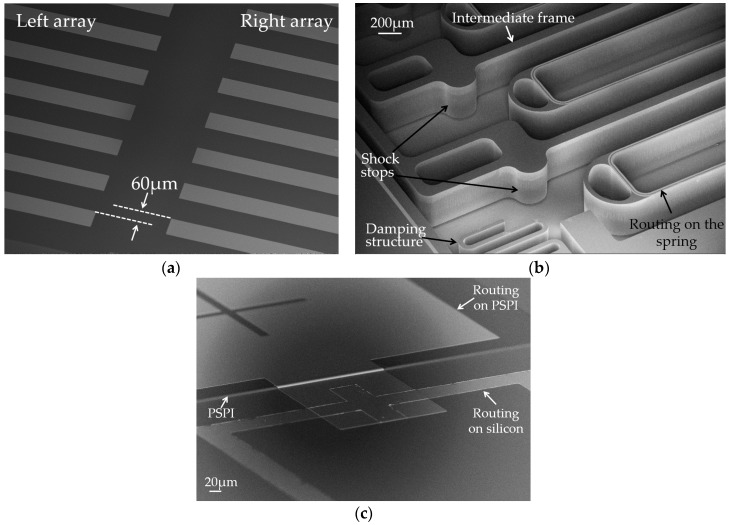
SEM image of some details on the proof-mass. (**a**) Periodic-sensing-array transducers; (**b**) details of routing, spring, damper and intermediate frames; (**c**) PSPI insulator and the routings on different layers.

Figure 8 presents the packaged MEMS, suspended proof-mass structure, top cap, and bottom cap. Some details are illustrated in Figure 9. In order to obtain a phase difference of π/2, the left and right arrays are deliberately staggered for 60 μm in the sensitive direction (Figure 9a). The intermediate frames and springs are also shown in Figure 9b. We can observe some shock stops and a damper structure, which are designed for protection purposes in case of overload. They are located at the corner of the external frame and at the edge of the intermediate frames. Figure 9c shows the SEM image of the PSPI insulator.

**Figure 10:**
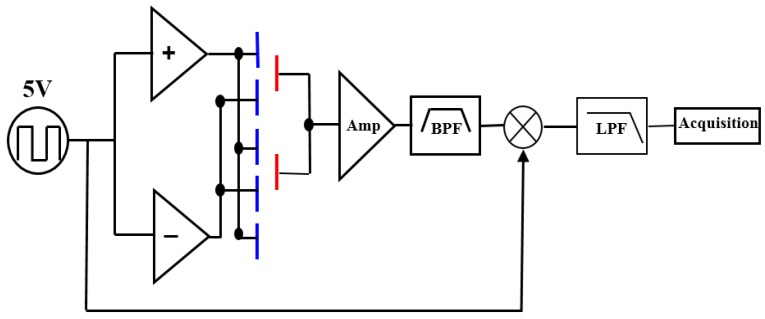
Block diagram of differential capacitance detection circuit.

**Figure 11:**
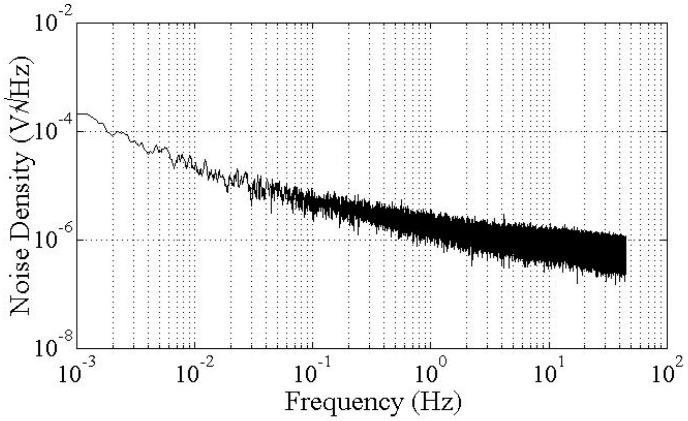
Noise power spectral density of our detection circuit.

The packaged accelerometer is connected to a printed circuit board (PCB) for resonant frequency and quality factor measurements. The circuit was developed in our earlier research [39,40], and the block diagram of the differential capacitive detection circuit is shown in Figure 10. A square wave, which has a zero-to-peak amplitude of 5 V and a frequency of 100 kHz, is used as carrier wave. By applying it on both right and left capacitive detection systems with a phase difference of π, the low-frequency signal of the differential capacitive output is modulated. Then, after the band-pass filtering and demodulation, the final differential data are acquired with a NI 6281 card. Figure 11 demonstrates the noise power spectral density of our detection circuit. It can be seen that the noise floor is 6 µV/√Hz at 0.1 Hz.

**Figure 12:**
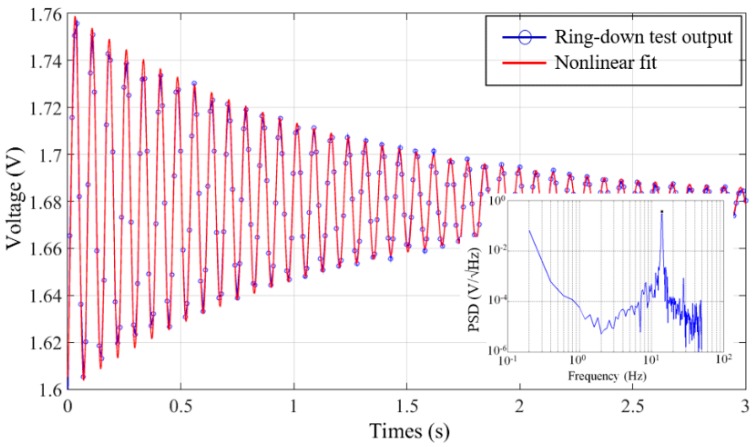
Ring-down test output of packaged MEMS accelerometer. Lower-right inset: power spectrum density (PSD) of ring-down test output.

Figure 12 shows the ring-down test output of the packaged MEMS accelerometer (blue line with circle) and the nonlinear fitting curve (red line). In the frequency domain, the power spectrum density (PSD) curves of the ring-down test output are shown in the lower-right inset. The results of nonlinear fitting show that the system has a quality factor of 47 at 1 atm with the resonance frequency of 13.2 Hz. The calculated mechanical noise equivalent acceleration is 1 ng/√Hz accordingly. The rejection ratio of the z-mode to the fundamental mode determined from SEM measurements is 6.7 (6.5 for the FEM simulation). These findings are consistent with the theoretical predictions as well as with ANSYS simulations.

**Figure 13:**
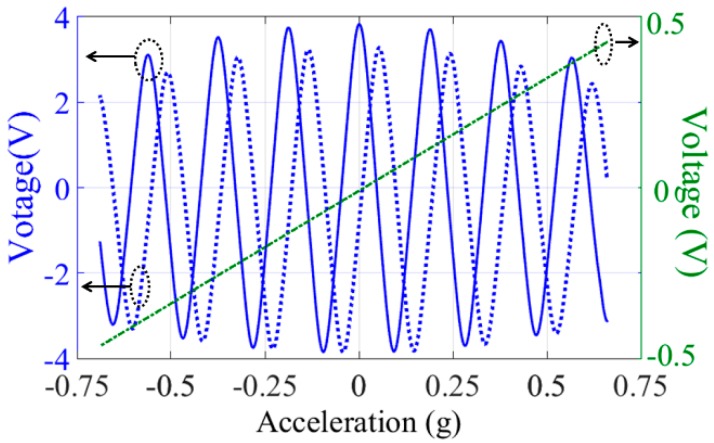
Differential output response to an acceleration range of ±0.7 g (measured under normal air pressure).

A commercial spring accelerometer is employed in the static calibration. Figure 13 shows the open-loop output response (calibration results) of our MEMS accelerometer to an acceleration variation about ±0.7 g, which is provided by tilting its sensitive axes in a local gravity field. As expected, we have successfully obtained a linear response and two sinusoidal signals. The linear response is given by the elementary capacitive pickup electrodes, which are used for a rough acceleration measurement at micro-g level in a large operating range. The measured sensitivity is, accordingly, about 0.26 pF/g. The measured system noise floor of elementary capacitive electrodes presents at a level of 10 μg/√Hz. The sinusoidal outputs are given by the left and right periodic-sensing-array transducers, which are used for extreme high resolution (nano-g) measurements in a small range around the balance point. The staggered left and right arrays guarantee that the inflection point of one periodic-sensing-array transducer output can be avoided by an alternate operation. Consequently, the measurement system could always work in the most sensitive areas. The sinusoidal outputs presented a capacitive sensitivity of 53 pF/g, and the corresponding sensitivity is 122 V/g. By combining the rough linear output and the precise sinusoidal outputs, our MEMS could provide a high-resolution measurement in the entire operating range. In addition, a dynamic measurement is also applied, and the MEMS could provide an excellent output as well. However, as shown in Figure 13, it is clearly seen that the magnitudes of the sinusoidal outputs are nonidentical. It is apparent that there is an envelope, and the maximum magnitude appears around zero input. As mentioned previously, although five intermediate frames are introduced in our design to increase the stiffness of the suspension along the *z*-axis, the proof mass will still have a vertical sag under gravity. By tilting its sensitive axes in a local gravity field, the *x*-axis component of the gravity that is parallel to the plane of proof-mass provides input acceleration for calibration purposes. Meanwhile, the vertical sag of the suspension is caused by the *z*-axis component of the gravity which is perpendicular to the plane of the proof-mass. Apparently, the vertical sag is not identical with different tilt angle, which leads to the variation in the maximum output of the sinusoidal signals.

**Figure 14:**
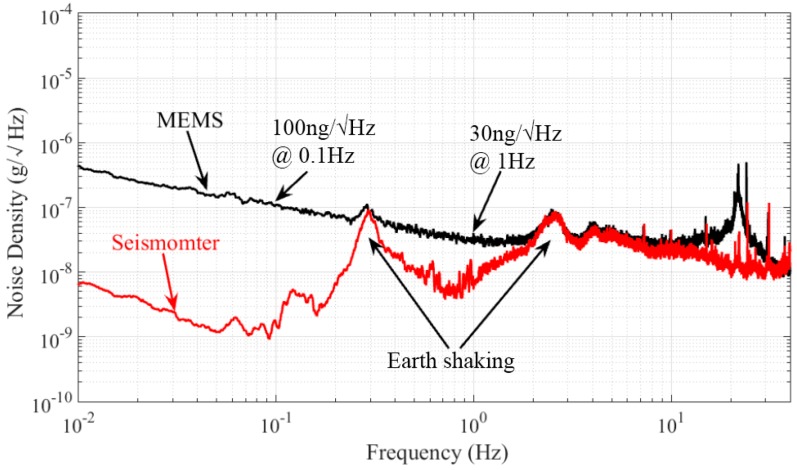
Periodic-sensing-array transducer output noise spectral density.

**Figure 15:**
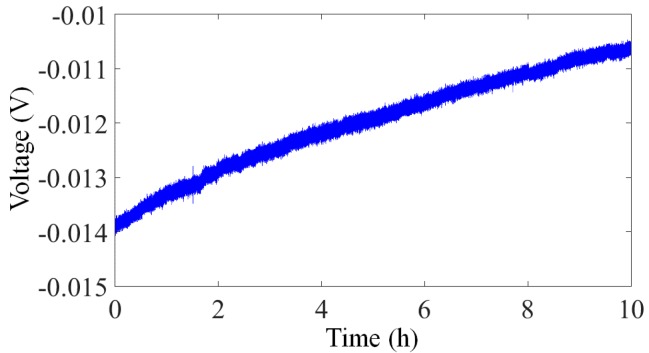
Zero drift of the accelerometer for 10 h at room temperature.

**Figure 16:**
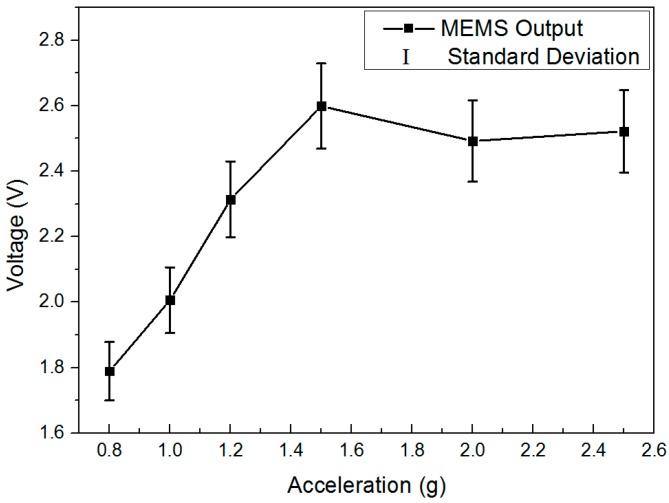
The output of MEMS accelerometer during test from ±0.8 g to ±2.6 g.

The noise power spectral density of the array transducer is demonstrated in Figure 14. The noise floor could be limited by the environmental disturbances. Therefore, the measurement was performed in our underground lab with a “quiet” environment by taking special care with additional ground mechanical decoupling, making it suitable for noise measurement down to 10 ng/√Hz [41]. Ten hours of static stable data are collected for this analysis. The system output noise floor is found to be 100 ng/√Hz at 0.1 Hz and 30 ng/√Hz at 1 Hz (1 atm). The peak of earth shaking is also detected by a commercial high-precision seismometer (CMG-3ESPC, Guralp, Aldermaston, UK), which indicates that the noise floor of our MEMS is almost the same as the background noise in our lab in this bandwidth. The presented noise floor density of the MEMS accelerometer system is similar to that of the PCB circuit. For a gravity gradient measurement application such as rotating accelerometer-based GGI, the operational frequency is usually from 0.1 Hz to 1 Hz [9]. Consequently, this capacitive in-plane nano-g MEMS accelerometer provides a good candidate for GGI applications. Figure 15 shows the zero drift of the accelerometer with the open-loop circuit for 10 h at room temperature. The bias stability is 30 μg. The reliability test is also performed by a dynamic shaker. The output is demonstrated in Figure 16. The full-scale output appears around ±1.4 g, which satisfies the operating range requirement for GGI applications. According to (1) and the measured resonant frequency, the maximum movement of proof mass can be estimated. The calculated value based on the measurement results is ±1.77 mm, which is smaller than that calculated based on the theoretical design (±2.37 mm). The difference is mainly caused by imprecise machining tolerance in microfabrication. More importantly, this error is acceptable for our final objective. As can be seen in the figure, this MEMS accelerometer is able to sustain dynamic shake up to ±2.6 g. No structural damages are found, and the MEMS could operate properly after the shock test.

**Figure 17:**
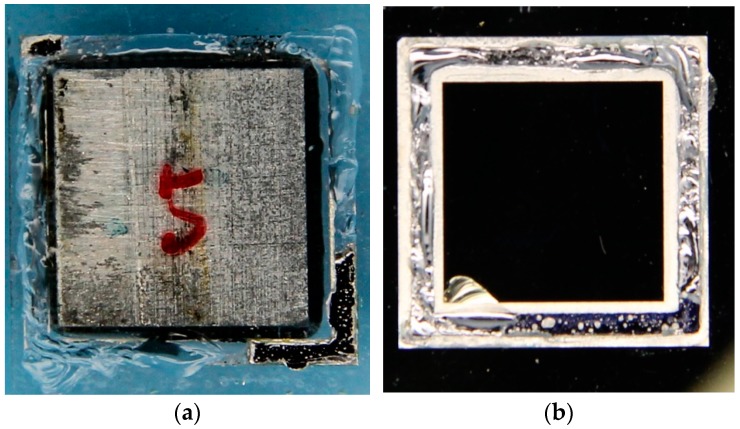
Photographs of fracture interface of a sample after tensile test. (**a**) Glass side; (**b**) silicon side.

The bonding strength of the SnPb solder bonding employed as the bonding technology for top-cap encapsulation has also been measured by a tensile test. Figure 17a,b show the typical photographs of the fracture surface of a sample pair. The average bonding strength was 5.0 MPa. Compared to the tensile strength (33.9 MPa) of the SnPb solder [42], the bonding strength presented here is significantly lower. However, it can be clearly observed that glass is torn apart from the glass substrate and transferred onto the bonding pattern of the silicon substrate. This fracture surface indicates that the tensile strength of the solder exceeds that of the glass, if the bonding is successfully achieved. The hermeticity of the sealed cavities in a seamlessly bonded area for the above three cases is analyzed and compared using the specifications prescribed by the MIL-STD-883E standard (method 1014.10) [43]. The hermeticity test includes a fine leak test and a gross leak test. The fine leak test consists of a helium over-pressure stage (known as “bombing”) followed by a helium leak test using a mass spectrometer. According to testing procedures stated in the aforementioned standard, the samples are first placed in a bombing chamber with helium gas at a pressure of 75 psia (∼0.52 MPa) for an exposure time of 3.5 h (helium bombing). Then the samples are unloaded from the bombing chamber and are subjected to a helium leak test using a mass spectrometer detector. The detection must be completed within the dwell time of 1 h defined as the time efflux between unloading from the bombing chamber and the end of the leak test. 80% of our samples present a leak rate smaller than the reject limit (5 × 10^−8^ atm·cm^3^/s) defined by the MIL-STD-883E standard [43], which demonstrates an outstanding bonding quality against harsh environments for hermetic encapsulation in 3D integration applications. Since the helium detector is used for a fine leak test for a leak rate below 10^−^^4^ atm·cm^3^/s, the samples need to be checked for the possibility of gross leaks. All unleaked samples under testing are subjected to a gross leak test using the standard bubble method with fluorocarbon liquids (FC-72, FC-43) according to the MIL-STD-883E standard (method 1014.10) [43]. Fluorocarbon FC-72 (boiling point: 60 °C) is used as the detector fluid, and fluorocarbon FC-43 (boiling point: 178 °C) is used as the indicator fluid. All of the samples under testing are immersed in FC-72 under a pressure of 75 psia for 3 h. They are then unloaded from the detector fluid and dried for 2 min. Finally, these samples are immersed in the indicator fluid FC-43 which is maintained at 125 °C. In our study, all unleaked samples successfully passed the gross leak test. No bubbles were observed from the sample during the test, even under a magnifying glass. The outstanding tensile test results and the hermeticity results show an excellent bonding quality against harsh environments for hermetic encapsulation in 3D integration applications.

## 5. Conclusions

In this paper, we have presented a design for an in-plane high-sensitivity MEMS accelerometer with elementary capacitive pickup electrodes and alternate periodic-sensing-array transducers. The accelerometer employs a simple sandwich structure, and it is fully CMOS compatible. A series of springs are used to increase the operating range. Intermediate frames are introduced to increase the rejection ratio of the spurious modes. In order to avoid collisions of the moving structures, shock stops and dampers are employed. The sensitivity and noise floor of our fabricated MEMS accelerometer are 122 V/g and 100 ng/√Hz (at 0.1 Hz), respectively. By combining the output of elementary capacitive pickup electrodes with periodic-sensing-array transducers, our MEMS could provide a nano-g resolution measurement in an operating range larger than ±1 g. It provides a dynamic range of over 140 dB from 0.1 Hz to 10 Hz. The measurement results demonstrate that this MEMS accelerometer could be further used in high-precision gravity gradient measurement applications.

## Figures and Tables

**Table 1:** Comparison of silicon’s mechanical properties to those of other materials.

Material	Young’s Modulus (GPa)	Yield Strength (Mpa)	Coefficient of Thermal Expansion 10^−6^ (/°C) at 20 °C
Single-Crystal Silicon <111>	190 [10]	6900 [10]	2.56 [11]
Stainless Steel	200 [12]	2100 [12]	11.9–18 [13]
Beryllium Bronze	128 [13]	400 [13]	17.8 [13]
Quartz	380 [10]	14000 [10]	0.5 [14]

**Table 2:** Physical dimensions of the accelerometer.

Symbol	Properties	Value
*L_Device_*	Device length	44.5 mm
*W_Device_*	Device width	32.2 mm
*L_Mass_*	Mass length	18 mm
*W_Device_*	Mass width	15 mm
*T*	Wafer thickness	500 μm
*m*	Weight of the proof mass	0.31 g
*w*	Width of springs	42 μm
*l*	Length of springs	8 mm
*w_s_*	Linkage width	100 μm
*W_frame_*	Width of the intermediate frames	100 μm
*n*	Number of springs	6
*n−1*	Number of intermediate frames	5
*k*	Spring constant	2.9 N/m
*f_r_*	Resonant frequency	15.2 Hz
*d*	Distance between the proof mass and top cap	20 μm
*L_elementary_*	Length of elementary electrodes	5.5 mm
*W_elementary_*	Width of elementary electrodes	1 mm
*L_array_*	Length of single array electrode	100 μm
*W_array_*	Width of single array electrode	3 mm
*n_array_*	Number of array electrodes on proof mass	45
